# Etymologia: **Lassa **[lah sə] **virus**

**DOI:** 10.3201/eid1606.ET1606

**Published:** 2010-06

This virus was named after the town of Lassa at the southern end of Lake Chad in northeastern Nigeria, where the first known patient, a nurse in a mission hospital, had lived and worked when she contracted this infection in 1969. The virus was discovered as part of a plan to identify unknown viruses from Africa by collecting serum specimens from patients with fevers of unknown origin. Lassa virus, transmitted by field rats, is endemic in West Africa, where it causes up to 300,000 infections and 5,000 deaths each year.
